# Death Due to Anaphylactic Reaction: The Role of the Forensic Pathologist in an Accurate Postmortem Diagnosis

**DOI:** 10.3390/medicina59122184

**Published:** 2023-12-15

**Authors:** Fabio Del Duca, Alice Chiara Manetti, Aniello Maiese, Gabriele Napoletano, Alessandro Ghamlouch, Natascha Pascale, Bolino Giorgio, Frati Paola, Raffaele La Russa

**Affiliations:** 1 Department of Anatomical, Histological, Forensic and Orthopaedic Sciences, Sapienza University of Rome, Viale Regina Elena 336, 00161 Rome, Italy; fabio.delduca@uniroma1.it (F.D.D.); alicechiara.manetti@uniroma1.it (A.C.M.); gabriele.napoletano@uniroma1.it (G.N.); alessandro.ghamlouch@uniroma1.it (A.G.); giorgio.bolino@uniroma1.it (B.G.); paola.frati@uniroma1.it (F.P.); 2 Department of Surgical Pathology, Medical, Molecular and Critical Area, Institute of Legal Medicine, University of Pisa, 56126 Pisa, Italy; 3 Department of Forensic Medicine, Hospital ‘San Carlo’, 85100 Potenza, Italy; nataschapascale@gmail.com; 4 Department of Clinical Medicine, Public Health, Life Sciences, and Environmental Sciences, University of L’Aquila, 67100 L’Aquila, Italy; raffaele.larussa@univaq.it

**Keywords:** anaphylaxis, anaphylactic-related death, legal medicine, forensic science, differential diagnosis, autopsy, immunohistochemistry

## Abstract

*Background and Objectives*: The diagnosis of anaphylaxis comprehensively depends on both situational information and laboratory investigations. For this purpose, serum tryptase concentration is examined as an indicator of systemic mast cell mediator release, linked to an underlying anaphylactic process. Increased levels of tryptase may occur in some events different from anaphylaxis, but usually information from crime scene investigations is lacking and autoptic findings are not specific. For legal reasons, it is required to achieve a definite diagnosis of mast cell degranulation that can lead to a certain diagnosis of death from anaphylaxis. Immunohistochemistry seems to be a relatively simple, reliable, and easily repeatable method that can assist the forensic pathologist in the differential diagnosis of death from anaphylaxis. *Materials and Methods*: This work provides an overview of the current literature on immunohistochemical methods useful in the determination process of anaphylactic-related deaths. A systematic search, according to the PRISMA statement, was performed in databases to identify studies investigating immunohistochemical targets related to anaphylaxis death. *Results*: This work underscores the importance of anaphylaxis mediators such as tryptase, CD117, and chymase in the immunohistochemical analysis of anaphylactic deaths. *Conclusions*: According to the reviewed literature, the diagnosis of death due to anaphylaxis should depend not just on the suspicion of an anaphylactic reaction but also on confirming mast cell degranulation through the identification of IHC positivity for inflammatory mediators, particularly in the respiratory tract.

## 1. Introduction

Anaphylaxis is an acute, life-threatening, systemic reaction caused by the immediate release of basophils and mast cells’ mediators in response to the exposure to an allergen [1]. It is characterized by various clinical presentations, but the most worrisome symptoms are the obstruction of the upper airway, bronchospasm, and hypotension [2]. The post-mortem diagnosis of fatal anaphylaxis may be challenging. Since death is usually the consequence of asphyxia or rapid shock, it could be difficult to identify useful post-mortem findings; in fact, there may not be sufficient time for specific features to appear. In the case of food, venom, or drug allergy, the specific features are generally present in only 59% of cases [3]. The suspicion of this kind of death arises when there is a history of poorly controlled asthma, insect bite, or medication/food intake immediately prior to collapse. In accordance with the Royal College of Pathologists’ guidelines, the pathologist has to carefully observe the body, looking for sites of stinging or biting by invertebrates, food debris, external signs of shock, and other features that may suggest a rapid mechanism of death. The autopsy could then provide more data, such as airway and lung edema. However, these features are typical but not specific; accordingly, they are not enough to sustain a diagnosis of anaphylactic reaction, especially if there is the hypothesis of a crime (i.e., suspected anaphylaxis due to food intake in a restaurant in a person who has well-known allergies).

An important role in the diagnosis of anaphylaxis is given to laboratory investigations. The serum tryptase concentrations, for example, have been studied as a marker of the release of great and systemic mast cells’ mediators. However, increased levels of tryptase may occur in some events other than anaphylaxis, such as multiple traumas, asphyxia, myocardial infarction, heroine intoxication, systemic mastocytosis, and hypereosinophilic parasitosis [4]. In addition, despite the lack of exhaustive studies on the real post-mortem stability of IgE immunoglobulins, some authors consider the assay on total and specific IgE serum levels (RAST, ELISA procedures) to be a reliable procedure applicable after death. The problem of the laboratory investigations is the intensity and speed of the cytolytic processes and the chemical degradation that could modify the concentration and stability of analytes.

Since autoptic and laboratory data may be absent or inconclusive, it is important to search for new methods to reach the post-mortem diagnosis of anaphylaxis death. Herein, we provide a systematic literature review to collect the current knowledge about the use of immunohistochemistry (IHC), evaluating the possibility of adding a new methodology that could help in the post-mortem diagnosis of anaphylaxis-related death.

## 2. Materials and Methods

**Figure 1:**
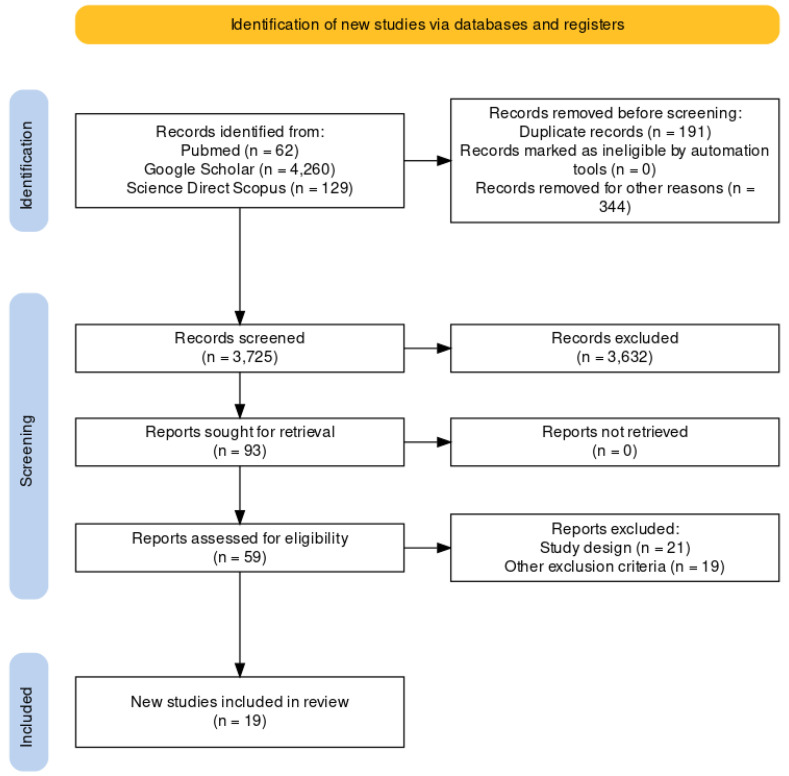
The selection of papers following PRISMA protocol.

The present systematic review was carried out according to the Preferred Reporting Items for Systematic Review (PRISMA) [5] standards. A methodological appraisal of each study was conducted according to the PRISMA standards, including an evaluation of bias. PRISMA 2020 Statement was applied. It consists of a checklist and a flow diagram (Figure 1).

A systematic literature search and critical review of the collected studies were conducted. An electronic search of PubMed, Science Direct Scopus, and Google Scholar from database inception to March 2023 was performed.

Databases were investigated using the following research terms (“anaphylaxis” OR “anaphylactic shock” OR “anaphylactic death” OR “tryptase” OR “anti-tryptase anti-body”) AND (“immunohistochemistry” OR “IHC” OR “immunofluorescence”) AND (“post-mortem” OR “autopsy” OR “forensic”) in all fields [e.g., title, abstract, and keywords].

From this research, a list of abstracts was organized in the form of a dataset and all of the dataset was downloaded in a .nbib file and uploaded to Software Zotero 6-0.30, used as a citation manager.

The research group, following a meeting, established the inclusion and exclusion criteria for the paper, in accordance with the PRISMA standard.

First of all, two investigators (AG and FDD) read all the abstracts found from databases. The bibliographies of all identified papers were examined and cross-referenced to further identify relevant literature.

After selecting abstracts and investigating the bibliographies of related papers, data collection began. One investigator (AG) independently examined papers with titles or abstracts that appeared to be relevant, selecting those that analyzed the use of IHC in anaphylactic death.

The data collection process included study selection and data extraction. Disagreements concerning eligibility among the researchers were resolved by consensus. Preprint articles were excluded, and only papers in English were included.

Data extraction was performed by two investigators (AG and FDD) and verified by additional investigators (AM, ACM, and FDD).

This study was exempt from institutional review board approval, as it did not involve human subjects.

## 3. Results

A review of the titles and abstracts, as well as a manual search of the reference lists, was carried out. The reference lists of all identified articles were reviewed to find missed literature. This search identified 93 articles, which were then screened based on their abstract. The resulting 93 papers were screened to exclude duplicates, which left 59 articles for further consideration. Non-English papers were excluded [n° 344], and the following inclusion criteria were used: (1) original research articles, (2) reviews and mini-reviews, and (3) case reports/series. These publications were carefully evaluated, considering the main aims of the review. This evaluation left 19 scientific papers comprising original research articles, case reports, and case series (see Figure 1).

We found articles about serum analytes (such as tryptase) and IHC with anti-tryptase antibody (AB), anti-CD117 and anti-chymase. Because of the specific aim of this work, only papers concerning IHC have been included in the systematic review. The selection of appropriate scientific papers was performed; nineteen articles met the inclusion criteria and were included.

### 3.1. The Study Sample

The study sample, considering all the scientific papers analyzed, comprised 162 cases of anaphylactic death (medium age 45.34—DS 17.24). In Table 1, we provide a summary of the main characteristics of the included studies, while Table 2 and Table 3 show the distribution of our studies per type of allergen.

In accordance with the different patterns of positivity of the ABs used to perform the IHC, the included cases have been divided into different groups. The anti-tryptase ABs provided positive results in all the cases; the anti-chymase ABs were positive in 24 up to 162 cases (14.8%) and anti-cd117 Abs were positive in 4/162 cases (2.5%).

Below, we provide a brief description of the immunohistochemical findings which we collected from the included studies, divided per type of AB.

### 3.2. Anti-Tryptase Antibody

The immunohistochemical pattern with anti-tryptase ABs shows a positivity in the lung tissue (see Table 4). Tryptase indicates the presence of mast cells. We found mast cells in the bronchial walls and in the vicinity of alveoli and capillaries; in some cases the connective interstitium and the pulmonary epithelial cells also resulted positive to these ABs. In the glottis and in the laryngeal wall, the presence of mast cells is also evident. In the minority of cases there were positive findings in myocardial tissue, spleen and stomach, jejunum intestinal tissue, and skin.

### 3.3. Anti-Chimase Antibody

These kinds of ABs present the same patterns as those of the anti-tryptase ABs in the pulmonary tissue with positivity at the site of the bronchial walls and in the vicinity of alveoli and capillaries (Table 5). Despite being less represented in all the cases, in some cases, they are present in the myocardial tissue and in the spleen.

### 3.4. Anti-CD117 Antibody

Anti-CD117 ABs are less represented than the anti-tryptase ones, but their pattern of positivity is similar (Table 6). Indeed, in the lung tissue, the typical shapes are starry-like and yard-like. In the laryngeal wall, beyond the typical pattern, we can find positivity in cells with dendritic morphology and in small lymphocytes. Moreover, there is positivity in the myocardium and in the intestinal mucosa.

## 4. Discussion

In this study, it was evident that antibiotics, cephalosporins above all, are the main cause of anaphylaxis; contrast agents and other drugs also have an important role. Considering all the 19 articles, in 9 of them the cause of the anaphylaxis was due to antibiotics. Blumentahl et al. underlined that adverse drug reactions account for more than 3% of hospital admissions and complicate the hospitalization of 10–20% of patients [25]. Antibiotics are the commonest cause of life-threatening immune-mediated drug reactions that are considered off target, including anaphylaxis, organ-specific adverse reactions, and severe cutaneous adverse reactions [26].

Our results show that ABs against tryptase, cd117, and chymase (mediators of anaphylaxis) are typically positive in cases of anaphylactic death, indicating the presence of mast cells. The tissue most frequently involved is the lung. Sometimes there is also a halo of positiveness around the cells after degranulation. On microscopical observation, we can find the typical starry-like and yard-like patterns.

Anaphylaxis is considered a systemic, life-threatening disorder triggered by mediators released by mast cells and basophils, activated via allergic (IgE-mediated) or nonallergic (non-IgE-mediated) mechanisms. Hypovolemia and distributive shock usually occur in severe anaphylaxis, and this is known by the historically termed “empty heart syndrome”. The incidence of anaphylaxis is probably underestimated. Publications from the last few years reveal an incidence of from 50 to 103 episodes per 100,000 persons/year. In the United States and United Kingdom, the mortality rate has been estimated to be less than 1 per million. According to the ICD-10, anaphylactic shock occurs as a response to allergen exposure (adverse food reaction, due to serum, adverse effect of drug or medication, possible Hymenoptera venom anaphylaxis events), which leads to the activation of mast cells and basophils, although in some cases, the cause remains unknown [27].

An analytical study of data in the US population shows that there were 2229 anaphylaxis-related deaths between 1999 and 2009 (0.69 per million population). The annual number of deaths ranged from 186 to 225, corresponding to mortality rates of between 0.63 and 0.76 per million. Of all anaphylaxis-related deaths, 75% were due to anaphylactic shock. Overall, 87% of the deaths occurred in a medical facility (inpatient, outpatient/ED, or dead on arrival), and approximately 7% occurred at home [28].

The typical triggers in IgE-mediated anaphylaxis are food; airborne allergens such as pollen, animal dander, aerosolized foods; latex; medications (oral or parenteral), food-dependent exercise-induced anaphylaxis; allergy to mammalian sugars such as galactose-1,3-alpha-galactose (alpha-gal); seminal fluid and hormones, radiocontrast media reactions [29,30], venom, and anesthetic drugs [31]. In the case of non-IgE-mediated anaphylaxis the typical triggers are IV immunoglobulins, NSAIDs (nonsteroidal anti-inflammatory drugs) [32], dialysis membranes, dextrans, iron, biological agents, and heparin.

According to LoVerde et al., clinically, the respiratory tract and the lungs are mostly implicated in cases of anaphylaxis (the typical finding is laryngeal edema). In fact, considering our data, we have noticed that all the antibodies are primarily positive in the lungs and in the laryngeal wall [33]. Death from anaphylaxis usually results from a combination of factors including upper airway obstruction from mucosal edema, asphyxia from bronchospasm, and shock due to massive fluid shifts [34].

**Figure 2:**
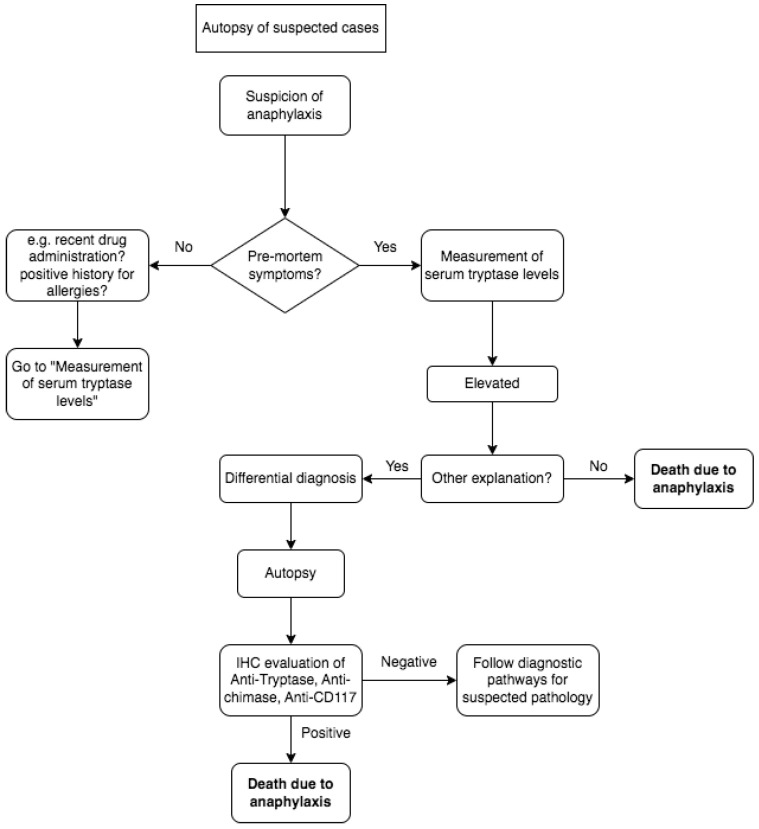
The diagnosis of anaphylaxis-related death in a suspected case is conducted by performing serum confirmation and/or immunohistochemical analysis, as shown in the flowchart.

In accordance with the main interpretation of anaphylaxis pathophysiology, mast cells and basophils are the primary effector cells in allergies, directly responding to allergen challenges through immunoglobulin-dependent or independent mechanisms. Upon activation, mast cells and basophils release three major groups of proinflammatory mediators, causing pathological damage that can lead to death. The lethal effects of allergies occur after the activation of mast cells or basophils. Theoretically, the identification of degranulation markers in mast cells and basophils is a definitive event in allergies, and finding them seems to be helpful in the post-mortem diagnosis of anaphylactic death [35,36]. Molecular methods of investigation could be helpful, as is the use of IHC. Our investigation reveals upper airway findings indicating positivity in the immunohistochemical evaluation of anti-tryptase, anti-chimase, and anti-CD117 Abs Tryptase which is consistently observed in all lung tissue samples, particularly in atopic asthmatic subjects, aiding in the identification of mast cells. Conversely, the presence of vacuolated mast cells appears to be indicative of degranulation in cases of anaphylaxis. Notably, certain samples exhibit anti-chymase-positive mast cells in perivascular spaces, displaying a similar pattern [8,14]. The assessment of the laryngeal wall and glottis includes an examination of the Tryptase and CD117 expression patterns, revealing the involvement of perivascular zones with vacuolized mast cells [12,23]. The diagnosis of anaphylaxis-related death is demanded in crime scene investigation, using information from anmnesis and circumstantial data, followed by tryptase serum dosage, and is eventually confirmed by immunohistochemical analysis, as shown in Figure 2.

In fact, as shown in the results, while anti-tryptase in lung tissues appears to be the most reliable marker, on the other hand, it is too general. So, as found in the literature review, an association with other markers such as anti-chymase and anti-CD117 is required to confirm the diagnosis. These two IHC markers of anaphylaxis seem to be not specific but have a good rate of sensitivity. Further studies are needed to confirm this hypothesis.

There are, however, numerous problems that arise in establishing the diagnosis of anaphylaxis at autopsy, as no typical macroscopic features are present in 41% of cases at autopsy. To corroborate the diagnosis, it could be useful to conduct a histological study of the laryngeal mucosa [3]. Moreover, to reach the diagnosis, an important role is given to the value of serum tryptase, which is considered the most widely used biomarker to confirm a diagnosis of anaphylaxis retrospectively [37]. On the other hand, there is the problem that serum histamine and tryptase levels are not always elevated [38], even in patients with severe manifestations of anaphylaxis including cutaneous, gastrointestinal, respiratory, or cardiovascular compromise [39].

A limit of the present study is the small sample size, due to the lack of articles about this topic. IHC has been relatively recently introduced in forensic investigations, and most of the included papers have been published in the last ten years. Moreover, this is a very targeted topic. Further studies are needed to evaluate how valid IHC could be in the demonstration of fatal anaphylactic shock.

## 5. Conclusions

Considering all the studies analyzed in this review and the fact that reaching a diagnosis of anaphylactic death could be an issue, IHC represents a fundamental tool for the pathologist. In fact, there are many cases where there is no possibility to perform laboratory tests in search for serum tryptase, or they are useless. Indeed, tryptase is not always elevated in anaphylaxis. Our work underlines the importance of some anaphylaxis mediators such as tryptase, cd117, and chymase in the immunohistochemical analysis in such kind of death. In accordance with the analyzed papers, the diagnosis of death due to anaphylaxis should be based not only on the incidence of anaphylactic reaction, but on the proof of the presence of these inflammatory mediators with IHC, especially in the respiratory tract. Even if further evidence needs to be collected, all the professionals involved in such investigations should be aware of the importance of IHC as a new reliable tool to reach the post-mortem diagnosis of anaphylaxis.

## Figures and Tables

**Table 1:** Main characteristics of the included studies.

References	no.	Age (Mean)	Sex	Molecule	Control Group no. (Cause of Death)	Sample
Fineschi et al. (2001) [6]	32	Not reported	Not reported	Anti-tryptase antibody	48 (heroin-related deaths) 44 (head trauma)	Lung
Edston et al. (1999) [7]	1	6–495 days	M (24) F (20)	Anti-tryptase antibody	N/A	Lung
Perskvist et al. (2007) [8]	9	17–55 (39)	M (7) F (1)	Anti-tryptase and anti-chymase antibody	20 (Asthma) 20 (Generic Natural causes)	Heart, Lung
Osawa et al. (2008) [9]	3	46–74 (58.6)	M (3)	Anti-tryptase and anti-chymase antibody	N/A	Lung
Turillazzi et al. (2008) [10]	1	33	F	Anti-tryptase antibody	N/A	Lung
Trani et al. (2008) [11]	4	53–65 (60)	M (3) F (1)	Anti-tryptase antibodies and anti-chymase antibody	N/A	Spleen
Unkrig et al. (2010) [12]	1	52	F	Anti-CD117 and anti-tryptase antibody	N/A	Glottis, Lung, Intestinal tissue
Luongo et al. (2011) [13]	2	30–47	M (2)	Anti-CD117 and anti-tryptase antibody	N/A	Glottis
Edston et al. (2013) [14]	8	Not reported	Not reported	Anti-tryptase and anti-chymase antibody	20 (Sudden Cardiac Death), 10 (Opiate-related death)	Lung, Spleen
Comment et al. (2014) [15]	6	19–68 (45.2)	M (72) F (22)	Anti-tryptase antibody	10 (hyperthermia), 10 (ketoacidosis), 10 (suicide), 18 (heroin overdose), 10 (sudden cardiac death), 10 (Acute myocardial infarct), 10 (car accident), 10 (arrhythmia)	Spleen
Bonetti et al. (2014) [16]	15	12–75 (55)	M (13) F (2)	Anti-tryptase antibody	40	Spleen
Guo XJ et al. (2015) [17]	15	Not reported	M (10) F (5)	Anti- tryptase, anti-carboxypeptidase A, anti-mouse IgG-TRITC Antibody	20	Stomach, Jejunum, Lung, Larynx, Heart
Radheshi et al. (2016) [18]	1	55	F	Anti-tryptase antibody	N/A	Spleen
Takahashi et al. (2016) [19]	1	60	F	Anti-mast cell tryptase antibody	1	Lung
Wang et al. (2020) [20]	20	0–75 (34.1)	M	Anti-human mast cell tryptase antibody, rabbit anti-human IgE antibody	52	Glottis, Lung, intestinal tissues
D’Errico (2020) [21]	1	79	M	Anti-tryptase antibody	N/A	Lung
Esposito et al. (2021) [22]	11	16–69 (47)	M (4) F (7)	Anti-tryptase monoclonal antibody	7 (sudden cardiac death), 4 (car accident),	Glottis, Lung, Skin
Tambuzzi et al. (2021) [23]	1	30	M	Anti-CD117 and anti-tryptase antibody	N/A	Glottis, Myocardiu, Lung
Feng et al. (2021) [24]	30	4–78 (45.23)	M (16) F (14)	Anti-FcεRIα and anti-tryptase antibody	30 (sudden cardiac death, falling from a height, or traffic accident)	Lung

Abbreviations: N/A: not applicable.

**Table 2:** Distribution of studies per type of allergen.

References	Food	Drugs	Illicit Drugs	Hymenoptera	Contrast Agent	Other
Fineschi et al. (2001) [6]	-	-	Heroin	-	-	-
Edston et al. (1999) [7]	-	-	-	-	-	Sudden infant death
Perskvist et al. (2007) [8]	Food	Penicillin-V	-	Mites, bee sting	Radio-opaque dye	Pollen, pets, unknown
Osawa et al. (2008) [9]	-	Nolport, Cefotiam hydrochloride	-	-	Ioxaglic acid	-
Turillazzi et al. (2008) [10]	-	-	-	-	-	Latex
Trani et al. (2008) [11]	-	Unasyn, Rocephin, Tazobac, Fidato	-	-	-	-
Unkrig et al. (2010) [12]	Food (unknown)	-	-	-	-	-
Luongo et al. (2011) [13]	-	Beta-lactam antibiotic, propolis	-	-	-	-
Edston et al. (2013) [14]	-	-	Opiates	-	-	-
Comment et al. (2014) [15]	-	-	-	-	Gadobutrol, iomeprol, iohexol	-
Bonetti et al. (2014) [16]	-	Penicillin, cephalosporin	-	Insect stings	Radiocontrast, gadolinium	-
Guo XJ et al. (2015) [17]	-	Penicillin, ceftriaxone, levofloxacina, lomefloxacina, ibuprofen	-	-	-	-
Radheshi et al. (2016) [18]	-	Clarithromycin	-	-	-	-
Takahashi et al. (2016) [19]	-	Ceftriaxone	-	-	-	-
Wang et al. (2020) [20]	-	Drug	-	-	-	-
D’Errico (2020) [21]	-	Ceftriaxone	-	-	-	-
Esposito et al. (2021) [22]	Food	Medications	-	-	Contrast medium injected	Latex
Tambuzzi et al. (2021) [23]	Peach	-	-	-	-	-
Feng et al. (2021) [24]	-	Ceftriaxone, safflower, azitromicina, ambroxol hydrochloride, sulbactam, cefoperazone, midazolam, cefuroxime, ceftazidime pentahydrate, Sanqi Panax Notoginseng for Injection	-	-	-	-

**Table 3:** Apten distribution shows a predominant role of drugs.

Type	Apten	no.
Food	Peach	1
	Unknown	2
Drugs	Penicillin	6
	Nolport	1
	Unasyn	1
	Cephalosporins	20
	Propolis	1
	Opiates	1
	Penicillin or cephalosporins	5
	Drug in general	26
	Levofloxacin	3
	Lomefloxacin	5
	Ibuprofen	1
	Clarithromycin	1
	Safflower	2
	Azithromycin	1
	Ambroxol	2
	Sulbactam and cefoperazone	10
	Sanqi Panax Notoginseng for Injection	1
	Midazolam	1
Illicit drugs	Heroin	48 (not all due to anaphylaxis)
Other	SIDS	44 (not all due to anaphylaxis)
	Latex	2
	Pollen	1
	Pets	1
	Unknown	1
Hymenoptera	Mites	1
	Bee sting	1
	Insects	4
Contrast agent	Radio-opaque dye	1
	Ioxaglic acid	1
	Gadobutrol	1
	Iomeprol	3
	Iohexol	2
	Gadolinium or radiocontrast	6
	Contrast medium injected	3

**Table 4:** Main findings per anti-tryptase.

References	Lung	Glottis/Laryngeal Wall	Myocardium	Spleen	Stomach, Jejunum Intestinal Tissues	Skin
Fineschi et al. (2001) [6]	Anti-tryptase antibodies used as a mast cell marker; they were found mostly in the lungs; occasionally tryptase-positive material was found outside but it is difficult to establish if the degranulation has taken place in vivo or during the processing of the tissue.	N/A	N/A	N/A	N/A	N/A
Edston et al. (1999) [7]	The anti-tryptase antibodies are positive in the lung tissue in accordance with the fact that prone position (pathogenic factor of SIDS) induces hypoxia and consequently the release of mast cell mediators. It could be also associated with a post-mortem artifact.	N/A	N/A	N/A	N/A	N/A
Perskvist et al. (2007) [8]	Anti-tryptase antibodies are positive in group-1 (anaphylactic death) lung sections in all the mast cell subsets at the site of bronchial smooth muscle and parenchymal tissues; group 2 (asthma associated death) also presents these patterns but with a smaller number of cells. The average number of eosinophil and mast cells is different between groups 1 and 2: 1:1 in the first one and 7:1 in the second one.	N/A	The amount of eosinophil and mast cells is higher only in group 1; in fact anaphylaxis is a systemic disorder while asthma is a local disorder.	N/A	N/A	N/A
Osawa et al. (2008) [9]	The anti-tryptase antibodies are positive in mast cells in the vicinity of alveoli and capillaries. (Differences were not statistically significant in terms of the tryptase-positive cells; in contrast, differences were meaningful with respect to the chymase-positive and doubly positive cells).	N/A	N/A	N/A	N/A	N/A
Turillazzi et al. (2008) [10]	Anti-tryptase anti-bodies in the bronchial walls and capillary septa, identifying pulmonary mast cells, and a great number of degranulated cells are identified with a halo of tryptase positiveness.	N/A	N/A	N/A	N/A	N/A
Trani et al. (2008) [11]	N/A	N/A	N/A	Mast cell positivity in splenic sinuses so that it could be useful to consider the spleen as a shock organ where the trigger is initially pressed by the allergen to start IAD (immediate anaphylactic death)	N/A	N/A
Unkrig et al. (2010) [12]	The antibodies are positive inside mast cells but also a vacuolar accumulation of anti-tryptase near mast cells is found and interpreted as a sign of degranulation; the typical patterns are starry sky-like and yard-like.	Throat mucosa mast cells (the same patterns of lungs).	N/A	N/A	Intestinal mucosa (the same patterns of lungs and throat).	N/A
Luongo et al. (2011) [13]	N/A	Anti-tryptase antibodies are positive in mast cells in the laryngeal wall.	N/A	N/A	N/A	N/A
Edston et al. (2013) [14]	The antibodies are positive in mast cells in the bronchial wall and the perivascular spaces (tryptase-positive mast cells are more numerous than chymase-positive ones).	N/A	N/A	Antibodies are positive in mast cells in the splenic red pulp (tryptase-positive mast cells are more numerous than chymase-positive ones).	N/A	N/A
Comment et al. (2014) [15]	N/A	N/A	N/A	Anti-tryptase antibodies highlight the presence of degranulated mast cells in spleen samples.	N/A	N/A
Bonetti et al. (2014) [16]	Anti-tryptase antibodies highlight mast cells’ degranulation in the interstitial space of the lungs.	N/A	N/A	Anti-tryptase antibodies show degranulated mast cells in spleen tissue.	N/A	N/A
Guo XJ et al. (2015) [17]	The expression of tryptase is evident in the bronchial walls and in the small vessel walls in the lung.	Tryptase is expressed in the small vessel walls in the submucosa of the larynx.	Tryptase is evident in the periphery mesenchyme of the small vessels in the heart.	N/A	Tryptase expression is evident in the mucous layer, and less in the muscular layer of the stomach and jejunum.	N/A
Radheshi et al. (2016) [18]	N/A	N/A	N/A	Anti-tryptase antibodies exhibit the characteristic degranulation of mast cells in spleen tissue.	N/A	N/A
Takahashi et al. (2016) [19]	Anti-tryptase antibodies show mast cells with a starry-sky pattern in the lung.	N/A	N/A	N/A	N/A	N/A
Wang et al. (2020) [20]	Anti-tryptase antibodies highlight a great number of mast cells mostly located around blood vessels and a few in pulmonary epithelial cells.	Anti-tryptase antibodies show mast cells in the laryngeal lamina propria around small blood vessels and cement glands.	N/A	N/A	Anti-tryptase antibodies are positive in the glands of the intestinal mucosa and in the connective tissue of the submucosa.	N/A
D’Errico (2020) [21]	Anti-tryptase antibodies highlight a great number of degranulating mast cells with tryptase-positive material outside.	N/A	N/A	N/A	N/A	N/A
Esposito et al. (2021) [22]	Anti-tryptase antibodies are found in the mast cells of the connective interstitium and bronchiolar structure.	The glottis shows an overexpression of anti-tryptase antibody at the vocal fold level.	N/A	N/A	N/A	Anti-tryptase antibodies highlight the presence of mast cells at the site of medication.
Tambuzzi et al. (2021) [23]	Antibodies highlight cells with cytoplasm rich in granules; the typical patterns are starry sky-like and yard-like.	Cells with cytoplasm rich in granules were documented in the glottis; additionally, there is the presence of lung patterns.	The positivity in the myocardium presents the same pattern as in the lung and glottis.	N/A	N/A	N/A
Feng et al. (2021) [24]	Anti-tryptase antibodies show mast cells, mainly located around bronchioles, bronchi, and blood vessels.	N/A	N/A	N/A	N/A	N/A

Abbreviations: N/A: not applicable.

**Table 5:** Main findings per anti-chimase.

References	Lung	Glottis/Laryngeal Wall	Myocardium	Spleen	Stomach, Jejunum Intestinal Tissues	Skin
Fineschi et al. (2001) [6]	N/A	N/A	N/A	N/A	N/A	N/A
Edston et al. (1999) [7]						
Perskvist et al. (2007) [8]	Anti-chymase antibodies highlight the same patterns of the anti-tryptase ones in all the mast cell subsets at the site of bronchial smooth muscle and parenchymal tissues.	N/A	Anti-chymase antibodies highlight heart mast cells, according to the fact that anaphylaxis is a systemic disorder.	N/A	N/A	N/A
Osawa et al. (2008) [9]	Pulmonary mast cells are in the vicinity of alveoli and capillaries (differences were not statistically significant in terms of the tryptase-positive cells; in contrast, differences were meaningful with respect to the chymase-positive and doubly positive cells).	N/A	N/A	N/A	N/A	N/A
Turillazzi et al. (2008) [10]	N/A	N/A	N/A	N/A	N/A	N/A
Trani et al. (2008) [11]	N/A	N/A	N/A	Mast cells have positivity in splenic sinuses so that it could be useful to consider the spleen as a shock organ where the trigger is initially pressed by the allergen to start IAD (immediate anaphylactic death)	N/A	N/A
Unkrig et al. (2010) [12]	N/A	N/A	N/A	N/A	N/A	N/A
Luongo et al. (2011) [13]						
Edston et al. (2013) [14]	The antibodies are positive in mast cells in the bronchial wall and the perivascular spaces (tryptase-positive mast cells are more numerous than chymase-positive ones).	N/A	N/A	Antibodies are positive in mast cells in the splenic red pulp (tryptase-positive mast cells are more numerous than chymase-positive ones).	N/A	N/A
Comment et al. (2014) [15]	N/A	N/A	N/A	N/A	N/A	N/A
Bonetti et al. (2014) [16]	N/A	N/A	N/A	N/A	N/A	N/A
Guo XJ et al. (2015) [17]	N/A	N/A	N/A	N/A	N/A	N/A
Radheshi et al. (2016) [18]	N/A	N/A	N/A	N/A	N/A	N/A
Takahashi et al. (2016) [19]	N/A	N/A	N/A	N/A	N/A	N/A
Wang et al. (2020) [20]	N/A	N/A	N/A	N/A	N/A	N/A
D’Errico (2020) [21]	N/A	N/A	N/A	N/A	N/A	N/A
Esposito et al. (2021) [22]	N/A	N/A	N/A	N/A	N/A	N/A
Tambuzzi et al. (2021) [23]	N/A	N/A	N/A	N/A	N/A	N/A
Feng et al. (2021) [24]	N/A	N/A	N/A	N/A	N/A	N/A

Abbreviations: N/A: not applicable.

**Table 6:** Main findings per anti-CD117.

References	Lung	Glottis/Laryngeal Wall	Myocardium	Spleen	Stomach, Jejunum Intestinal Tissues	Skin
Fineschi et al. (2001) [6]	N/A	N/A	N/A	N/A	N/A	N/A
Edston et al. (1999) [7]	N/A	N/A	N/A	N/A	N/A	N/A
Perskvist et al. (2007) [8]	N/A	N/A	N/A	N/A	N/A	N/A
Osawa et al. (2008) [9]	N/A	N/A	N/A	N/A	N/A	N/A
Turillazzi et al. (2008) [10]	N/A	N/A	N/A	N/A	N/A	N/A
Trani et al. (2008) [11]	N/A	N/A	N/A	N/A	N/A	N/A
Unkrig et al. (2010) [12]	The antibodies are positive inside mast cells but also a vacuolar accumulation of anti-tryptase near mast cells is found and interpreted as a sign of degranulation; the typical patterns are starry sky-like and yard-like.	Throat mucosa mast cells (the same patterns of lungs).	N/A	N/A	Intestinal mucosa (the same patterns of lungs and throat).	N/A
Luongo et al. (2011) [13]	N/A	Anti-cd117 antibodies are positive in mast cells in the laryngeal wall; moreover, cells with dendritic morphology and small lymphocytes are positive.	N/A	N/A	N/A	N/A
Edston et al. (2013) [14]	N/A	N/A	N/A	N/A	N/A	N/A
Comment et al. (2014) [15]	N/A	N/A	N/A	N/A	N/A	N/A
Bonetti et al. (2014) [16]	N/A	N/A	N/A	N/A	N/A	N/A
Guo XJ et al. (2015) [17]	N/A	N/A	N/A	N/A	N/A	N/A
Radheshi et al. (2016) [18]	N/A	N/A	N/A	N/A	N/A	N/A
Takahashi et al. (2016) [19]	N/A	N/A	N/A	N/A	N/A	N/A
Wang et al. (2020) [20]	N/A	N/A	N/A	N/A	N/A	N/A
D’Errico (2020) [21]	N/A	N/A	N/A	N/A	N/A	N/A
Esposito et al. (2021) [22]	N/A	N/A	N/A	N/A	N/A	N/A
Tambuzzi et al. (2021) [23]	Antibodies highlight cells with cytoplasm rich in granules; the typical patterns are starry sky-like and yard-like.	Cells with cytoplasm rich in granules were documented in the glottis (right and left vocal cords)	The positivity in the myocardium presents the same pattern as that of the lung and glottis.	N/A	N/A	N/A
Feng et al. (2021) [24]	N/A	N/A	N/A	N/A	N/A	N/A

Abbreviations: N/A: not applicable

## Data Availability

The data that support the findings of this study are available from the corresponding author upon reasonable request.
